# Interpreting the Emerging Discourse Around Elderhood: Life Stage, Anti-Ageism Strategy, or Something Else?

**DOI:** 10.1093/geroni/igab046.1915

**Published:** 2021-12-17

**Authors:** Jenny Inker

## Abstract

The emerging discourse around elderhood hints at the possibility of a later life stage characterized by a focus on balancing development and decline, with potential to empower elders. However, little agreement exists about whether elderhood is a valid and useful construct. The first presenter questions the aging “mystique” through an analysis of the concepts of elderhood, sageing, croning, and eldering in popular and academic literature, underscoring the importance of avoiding othering and critically thinking beyond labels, even if positive. The second presenter explores the concept of agency in later life through a feminist philosophical lens, arguing that confrontations with one’s existential vulnerability need not be an obstacle to agency in elderhood, but rather can inspire alternative conceptualizations of it. The third presenter contrasts his personal and professional experiences of studying cultural aspects of aging, concluding that elderhood is neither a stage of a life nor a rite of passage but rather an individual, voluntaristic process. The fourth presenter explores 943 texts written by Finnish older adults, finding that the writers creatively position themselves as a group of older persons with a special contribution to make to society, even where elderhood is not explicitly mentioned, and potentially offer an alternative view to countering ageism. The fifth and final presenter explores a novel elderhood video intervention among first-year medical students (N = 585). Thematic findings of neutrality, elderhood as development, elderhood as othering, and elderhood as an opportunity to reframe stigma suggest that elderhood may be a viable and productive anti-ageism strategy.

